# Comprehensive dietary antioxidant index and chronic kidney disease: mediating role of frailty and its impact on mortality outcomes in adults

**DOI:** 10.3389/fnut.2025.1679774

**Published:** 2025-10-14

**Authors:** Wenqing Li, Shuang Chen, Jiangmin Wan, Liyuan Chen, Lingzhi Xing, Zhenglan Gao, Ling Chen

**Affiliations:** ^1^Faculty of Pediatrics, Chongqing Medical University, Chongqing, China; ^2^The Center of Experimental Teaching Management, Chongqing Medical University, Chongqing, China; ^3^Department of Pain Treatment, Sichuan Tianfu New Area People's Hospital, Chengdu, China; ^4^Chongqing Hospital of Jiangsu Province Hospital, Chongqing, China

**Keywords:** antioxidant, chronic kidney disease, frailty, mediation analysis, mortality

## Abstract

**Background:**

Chronic kidney disease (CKD) poses significant global health challenges, with oxidative stress and inflammation contributing to its pathogenesis. While dietary antioxidants may mitigate CKD risk, reflected by Comprehensive Dietary Antioxidant Index (CDAI), the mediating role of frailty (FI) remains underexplored. This study investigates the association between CDAI and CKD risk, with a focus on FI as a potential mediator and its implications for mortality outcomes.

**Methods:**

Utilizing data from 11,904 U. S. adults in the National Health and Nutrition Examination Survey (NHANES, 2011–2018), we analyzed CDAI (comprising manganese, selenium, zinc, and vitamins A, C, E) and its association with CKD. Multivariable logistic regression, restricted cubic splines, and Cox proportional hazards models assessed relationships between CDAI, FI, CKD, and mortality. Mediation analysis quantified FI’s role in CDAI-CKD associations.

**Results:**

Higher CDAI scores were inversely associated with CKD prevalence (OR = 0.802, 95%CI [0.753, 0.854], *p* < 0.001), with a 46.6% lower CKD risk in the highest vs. lowest CDAI quartile. Frailty mediated 36% (95% CI: 35–38%) of the CDAI-CKD relationship. Manganese and vitamin C exhibited independent protective effects against CKD (*p* < 0.001). Survival analyses revealed lower CDAI correlated with higher all-cause mortality in pre-frail CKD patients (*p* = 0.030) and elevated cardiovascular mortality in frail patients (*p* < 0.0001). Vitamin E inversely linked to cardiovascular mortality (HR = 0.934, *p* = 0.019), while vitamin A increased risk (HR = 1.266, *p* = 0.005).

**Conclusion:**

Comprehensive Dietary Antioxidant Index is inversely associated with CKD risk, partially mediated by FI. Dietary antioxidant intake, particularly vitamins C and E, may improve outcomes in CKD populations, especially those with frailty. These findings highlight the potential of nutritional interventions to mitigate CKD progression and mortality. Further randomized trials are needed to confirm causality and optimize dietary strategies for high-risk groups.

## 1 Introduction

Chronic kidney disease (CKD) represents a global health challenge, affecting over 800 million people worldwide, with a prevalence exceeding 10% in the general population1 above (1). Patients with CKD face a substantially elevated risk of cardiovascular disease (CVD) and all-cause mortality, with approximately 50% of CKD patients dying from CVD (2, 3). Although oxidative stress and inflammation are well recognized as core drivers in the development and progression of CKD (4), effective nutritional intervention strategies to delay CKD progression and improve prognosis remain lacking. Consequently, CKD poses unique challenges to public health, particularly given its often asymptomatic early stages, which underscores the critical importance of early detection and management.

Recent studies have emphasized the role of oxidative stress and inflammation in the pathogenesis of CKD. Oxidative stress arises from an imbalance between reactive oxygen species (ROS) and antioxidants in the body, which can cause renal damage and exacerbate CKD (5, 6). Against this backdrop, dietary antioxidants are believed to exert a protective effect by alleviating oxidative damage and improving overall health outcomes. The Comprehensive Dietary Antioxidant Index (CDAI) is a composite measure for evaluating an individual’s overall exposure to dietary antioxidants. It is calculated based on multiple dietary antioxidants, including manganese, selenium, zinc, as well as vitamins A, C, and E (7, 8). Therefore, we hypothesize that an individual’s CDAI may, to some extent, reflect the degree of their renal damage. Recent studies have shown that CDAI is associated with various health outcomes, such as lower CVD mortality and all-cause mortality (9). However, there is a relative paucity of research on the relationship between CDAI and the development and progression of CKD, particularly regarding whether CDAI can serve as a valid predictor of adverse prognosis in CKD patients.

Meanwhile, CDAI is closely associated with frailty status, and this association may be partially mediated by oxidative stress (10). Frailty, a clinical syndrome characterized by reduced physiological reserve across multiple systems, is highly prevalent among CKD patients (11, 12). Studies have demonstrated that over 50% of CKD patients exhibit frailty or pre-frailty, and frailty is significantly associated with increased hospitalization rates, disability rates, and mortality in CKD patients (13–15). Frailty and CKD share multiple pathophysiological mechanisms, including oxidative stress, chronic inflammation, and metabolic disorders (16), which further accelerate the progression of adverse prognosis in patients. Thus, we hypothesize that CDAI may reduce CKD risk and improve its prognosis by alleviating oxidative stress and inflammatory status, thereby mitigating frailty.

This study aims to explore the relationship between CDAI and the risk of CKD using large-scale data from the National Health and Nutrition Examination Survey (NHANES), and to validate, for the first time, the mediating role of frailty in this relationship. Furthermore, we analyze the predictive value of CDAI and its components for all-cause mortality and cardiovascular mortality in CKD patients with different frailty statuses, with the goal of providing new theoretical basis and clinical strategies for nutritional intervention in CKD patients.

## 2 Methods

### 2.1 Study population

**Figure 1:**
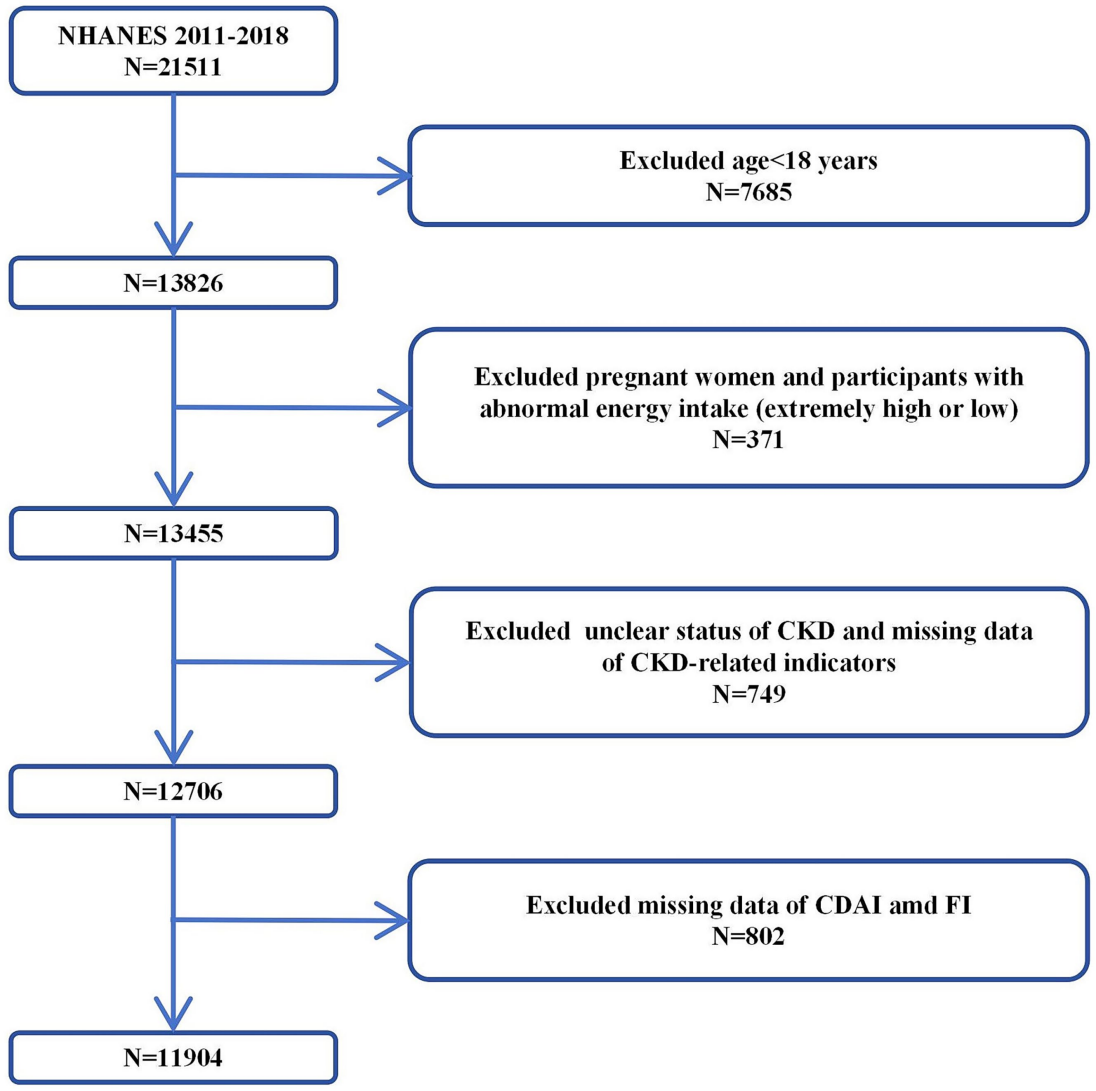
Selection process of the study population.

The NHANES is a series of cross-sectional surveys representing the non-institutionalized civilian population of the United States^1^. The NHANES includes demographic, socioeconomic, dietary, and health related questionnaire data collected through face-to-face interviews, physical and physiological examinations, and extensive laboratory tests. Due to deficiencies in data collection and preservation norms in the early period (1999–2010), there was a large amount of missing baseline data of the population in this period, as well as dietary survey and health detection data which are crucial for calculating CDAI and frailty index (FI). After comprehensive data cleaning, the valid data retained from 1999 to 2010 were insufficient to support reliable analysis. Therefore, we performed inclusion and exclusion from 21,511 participants in NHANES 2011–2018. Our inclusion criteria were: (1) complete data on indicators related to the calculation of CDAI and FI; (2) complete data on CKD-related indicators. The exclusion criteria were: (1) abnormal energy intake; (2) pregnancy status; (3) comorbidities that may interfere with CKD diagnosis (e.g., acute kidney injury). First, we excluded 7,685 participants under 18 years old, then excluded 371 pregnant women and participants with abnormal energy intake (extremely high/low). Next, we excluded 749 participants with unclear status of CKD and CKD-related indicators. Finally, 802 participants with missing CDAI and FI data were excluded. Therefore, a total of 11,904 participants was included in this study (Figure 1).

### 2.2 Exposure and outcomes

In NHANES, the food and nutrient intake of each participant is recorded through 24-h dietary recall interviews. The Food and Nutrient Database for Dietary Studies of the United States Department of Agriculture was used to calculate the intake of antioxidants, micronutrients, and total energy (17). Based on the questionnaire survey, we determined each participant’s dietary supplement intake over the past month, including dosage, frequency, and number of doses (18). To estimate the CDAI, we standardized each of the same six dietary vitamins and minerals by subtracting the global average and dividing by the global standard deviation. We then calculated the CDAI by adding the standard intake of these vitamins and minerals as follows. The CDAI is generally converted into categorical variables according to quartiles: Q1 < −2.27, −2.27 ≤ Q2 < −0.47, −0.47 ≤ Q3 < 1.17, Q4 ≥ 1.71.


$$\text{CDAI}=\sum_{i=1}^{n=6}\frac{\text{Individual Intake}-\text{Mean}}{\mathrm{SD}}$$

The CKD was identified at a urine albumin to creatinine ratio (UACR) ≥ 30 mg g − 1 and/or an estimated glomerular filtration rate (eGFR) < 60 mL per min per 1.73 m^2^ (19). The urine albumin/creatinine ratio was used to compute the UACR. The eGFR was calculated using the Chronic Kidney Disease Epidemiology Collaboration algorithm.

### 2.3 Assessment of frailty

Frailty was evaluated by the FI which was calculated by the accumulation of multiple age-related health deficits. In this study, we constructed the FI following standard procedures described previously (20–22). Based on the data of NHANES, we selected 9 items for FI construction, involving disease (excluding kidney disease and diabetes mellitus), symptom, physical function, and mental health (see Supplementary Table S1). Items 1–7 were dichotomized as 1 (presence of the deficit) or 0 (absence of the deficit) according to corresponding cut-off values, respectively. The item 8 and 9 (days of poor physical health per month, days of poor mental health per month) were a continuous variable which ranged from 0 to 30. We define a “poor day count” of ≤15 as 0 (indicating good condition) and >15 as 1 (indicating poor condition). For each participant, FI was calculated by the sum of the present health deficits divided by the number of items. Thus, the FI was a continuous variable ranging from 0 to 1, and a higher FI indicated a higher level of frailty. As suggested by previous studies (10, 23, 24), frailty status was classified into three categories: robust (FI ≤ 0.10), pre-frail (0.10 < FI < 0.25), and frail (FI ≥ 0.25).

### 2.4 Covariates

Information about covariates was obtained using baseline questionnaires. These questionnaires included questions about age, gender, education level, marital status, smoking status, poverty income ratio (ratio of family income to poverty threshold [PIR]), body mass index (BMI), waist circumference (WC), systolic blood pressure (SBP), Diastolic blood pressure (DBP), and self-reported baseline medical history such as DM and CKD. BMI was computed by measuring the height and weight. Urine creatinine (UC), urine albumin (UA), triglyceride (TG), glycated hemoglobin a1c (HbA1c), serum creatinine (SCR), Serum Uric Acid (SUA) and high-density lipoprotein cholesterol (HDL-C) were included in the biochemical profile.

We divided educational background into less than high school (less than 9th grade or 9–11th grade [including 12th grade with no diploma]), high school or equivalent, and more than high school (some college or associate’s degree or college graduate or above). The marital status was classified as married/widowed/divorced/separated/never married/living with a partner.

Hypertension was defined as average systolic blood pressure (SBP) ≥ 140 mmHg and/or average diastolic blood pressure (DBP) ≥ 90 mmHg; or self-reported diagnosis of hypertension and intake of antihypertensive medications. Diabetes mellitus (DM) was defined as (1) doctor diagnosed diabetes; (2) glycohemoglobin >6.5%; (3) fasting glucose ≥7.0 mmol/L; (4) random blood glucose ≥11.1 mmol/L; (5) two-hour oral glucose tolerance test blood glucose ≥11.1 mmol/L; and (6) use of diabetes medication or insulin (18). The full measurement technique for these variables is available at https://www.cdc.gov/nchs/nhanes/.

The missing rates of covariates were summarized in Supplementary Table S2. The missing data of covariates were imputed using the multiple imputation with chained equation.

### 2.5 Statistical analyses

Based on the “minimum sub-sample principle” and NHANES guidelines: when the data includes 24-h dietary recall and involves multi-day recall, the weight corresponding to the day with the smallest sub-sample size is selected. We used the 24-h dietary recall weight (specifically, wtdrd2 for the second day of 24-h dietary recall) to standardize our data to ensure representativeness.

For descriptive statistics, continuous variables were expressed as mean [standard deviation ±SD] or median [interquartile range ±IQR], while categorical variables were expressed as number (percentage).

To analyze the association of CDAI with the risks of incident CKD, a multivariate linear regression model was used. The CDAI is generally converted into categorical variables according to quartiles, and the *p*-values of the trend are calculated. Three models are used in this study. Model 1 was a crude model not adjusted for potential confounding factors. Model 2 was adjusted for age, gender, SBP, and DBP. Model 3 was further adjusted for PIR, drinking status, and HbA1c. We also assessed the presence of non-linear dose–response relationships between the risks of CKD and CDAI levels or the components of CDAI (Manganese, Selenium, Zinc, Vitamins A, Vitamins C, Vitamins E), respectively, using restricted cubic splines (RCS).

To analyze the association of frailty status with the risks of incident CKD, Cox proportional hazard regression was used to calculate the hazard ratio (HR) and its 95% confidence interval (95% CI). Three models were fitted for the Cox regression using robust participants as the reference. Model 1 was unadjusted. Model 2 was adjusted for age, gender. Model 3 was further multivariable-adjusted controlling for age, gender, HbA1c, Serum Creatinine, Serum Uric acid.

Causal mediation analysis was conducted to investigate whether FI increase mediates the associations of CDAI with CKD risk. Based on the criteria of the mediator, in addition to the negative association of X (CDAI) with Y (CKD risk), statistically significant associations of X with M (FI), M with Y should be observed. Therefore, we additionally performed generalized linear model (GLM) to examine the associations of CDAI with FI, FI with CKD risk. If these criteria were met, the mediation effect model would be performed to evaluate the mediated proportion (%) of FI in the relationships between CDAI with CKD risk.

Kaplan–Meier (KM) curves were used to show different survival patterns among the CKD population with different levels of CDAI in different FI status. We also employed the Cox proportional hazards regression analysis to investigate the correlation between different survival patterns and components of CDAI (manganese, selenium, zinc, vitamin A, vitamin C, vitamin E) in the CKD population.

All statistical analyses were carried out by IBM SPSS Statistics 27 and R software (Version 4.3.3). Statistical significance was set at *p* < 0.05.

## 3 Result

### 3.1 Baseline characteristics of the study population

A total of 11,904 participants (female: 50.6%, mean age: 49.73 ± 17.54 years) were included in the baseline analyses. The baseline characteristics of these participants are presented in Table 1. Frail participants were older, more likely to be female, less likely to be never married or living with partner, and had lower PIR than robust participants. In terms of the disease, the baseline levels of underlying diseases (hypertension, DM, and CKD) are not balanced among the Robust, Pre-frail, and Frail groups, and all indicators have a *P*-value less than 0.001, indicating significant differences. Frail participants also had higher levels of BMI, WC, SBP, UA, HbA1c, SCR, SUA, TG, ACR, and a lower level of DBP, Mn, Zn, VE and eGFR as compared with robust participants. What’s more, the CDAI scores demonstrate an approximate normal distribution, while the FI is concentrated at lower levels (Supplementary Figure S1).

**Table 1:** Baseline characteristics of participants for baseline frailty status analyses.

Variables	NHANES (*n* = 11,904)				
	Total (*n* = 11,904)	Robust (*n* = 4,748)	Pre-frail (*n* = 3,482)	Frail (*n* = 3,674)	*P*-value
Clinical characteristics					
Age	49.73 ± 17.54	39.82 ± 14.79	50.85 ± 16.36	61.47 ± 13.97	<0.001
Sex					<0.001
Male	5,880(49.40)	2,454(41.70)	1760(29.90)	1,666(28.30)	
Female	6,024(50.60)	2,294(38.10)	1722(28.60)	2008(33.30)	
PIR	2.54 ± 1.62	2.69 ± 1.64	2.57 ± 1.64	2.33 ± 1.56	<0.001
Education					<0.001
Less than high school	992(20.9)	172(26.1)	310(47.1)	176(26.7)	
High school or equivalent	2,393(50.4)	261(26.1)	486(48.6)	253(25.3)	
More than high school	1,363(28.7)	559(18.1)	1,597(51.7)	934(30.2)	
Marriage					<0.001
Married	5,984(50.30)	2,348(39.20)	1778(29.70)	1858(31.00)	
Widowed	845(7.10)	99(11.70)	228(27.00)	518(61.30)	
Divorced	1,320(11.10)	363(27.50)	409(31.00)	548(41.50)	
Separated	398(3.30)	107(26.90)	139(34.9)	152(38.2)	
Never married	2,329(19.60)	1,339(57.50)	588(25.20)	402(17.30)	
Living with partner	1,028(8.60)	492(47.90)	340(33.10)	196(19.10)	
Drinking status					<0.001
Never drinkers	9,583(80.50)	3,756(39.20)	2,729(28.50)	3,098(32.30)	
Ever drinkers	2,321(19.50)	992(42.70)	753(32.40)	576(24.80)	
Hypertension					<0.001
Suffer from hypertension	2,612(21.94)	488(10.28)	855(24.55)	1,269(34.54)	
Free from hypertension	9,292(78.06)	4,260(89.72)	2,627(75.45)	2,405(65.46)	
DM					<0.001
Suffer from DM	2,288(19.20)	313(6.60)	697(20.00)	1,278(34.80)	
Free from DM	9,616(80.80)	4,435(93.40)	2,785(80.00)	2,396(65.20)	
CKD					<0.001
Suffer from CKD	1,220(10.20)	104(2.20)	333(9.60)	783(21.3)	
Free from CKD	10,684(89.80)	4,644(97.80)	3,149(90.40)	2,891(78.7)	
BMI	29.45 ± 7.09	27.58 ± 6.14	29.85 ± 6.97	31.49 ± 7.69	<0.001
WC	100.16 ± 16.91	94.35 ± 15.24	101.35 ± 16.24	106.56 ± 17.05	<0.001
SBP	125.01 ± 08.42	118.19 ± 14.68	126.72 ± 18.28	132.20 ± 19.74	<0.001
DBP	71.08 ± 12.88	70.38 ± 11.45	72.01 ± 13.01	71.09 ± 14.38	<0.001
Laboratory data					
Mn	0.04 ± 0.02	0.05 ± 0.02	0.04 ± 0.02	0.03 ± 0.02	<0.001
Se	0.11 ± 0.06	0.12 ± 0.07	0.11 ± 0.06	0.11 ± 0.06	<0.001
Zn	10.68 ± 6.87	11.26 ± 7.16	10.56 ± 6.90	10.04 ± 6.39	<0.001
VA	0.61 ± 0.69	0.62 ± 0.67	0.60 ± 0.74	0.61 ± 0.65	0.320
VC	82.54 ± 94.85	85.45 ± 94.94	84.09 ± 105.11	77.32 ± 83.64	<0.001
VE	8.50 ± 6.74	8.88 ± 6.70	8.50 ± 7.29	8.02 ± 6.18	<0.001
UCR	124.08 ± 82.36	127.87 ± 85.89	125.70 ± 81.53	117.65 ± 78.02	<0.001
UA	46.10 ± 287.17	20.02 ± 155.62	42.55 ± 250.22	83.17 ± 417.54	<0.001
HbA1c	5.80 ± 1.09	5.48 ± 0.77	5.86 ± 1.14	6.14 ± 1.26	<0.001
SCR	0.90 ± 0.39	0.85 ± 0.23	0.89 ± 0.41	0.98 ± 0.50	<0.001
SUA	5.45 ± 1.45	5.24 ± 1.34	5.48 ± 1.44	5.71 ± 1.54	<0.001
TG	150.46 ± 122.30	136.83 ± 115.26	156.78 ± 127.46	162.09 ± 124.48	<0.001
HDL-C	53.08 ± 15.84	53.88 ± 15.30	52.63 ± 15.95	52.46 ± 16.36	0.01
ACR	0.46 ± 3.12	0.17 ± 1.37	0.45 ± 3.29	0.84 ± 4.30	<0.001
eGFR	90.86 ± 23.24	100.75 ± 19.06	90.55 ± 22.13	78.38 ± 23.12	<0.001
CDAI	0.06 ± 3.54	0.41 ± 3.53	0.04 ± 3.69	−0.37 ± 3.34	<0.001

NHANES, National Health and Nutrition Examination Survey; PIR, Price-to-Income Ratio; DM, diabetes mellitus; CKD, chronic kidney disease; BMI, body mass index; WC, waist circumference; SBP, systolic blood pressure; DBP, diastolic blood pressure; VA, vitamin A; VC, vitamin C; VE, vitamin E; UCR, urine creatinine; UA, urine albumin; HbA1c, glycated hemoglobin A1c; SCR, serum creatinine; SUA, serum uric acid; TG, triglyceride; HDL-C, high lipoprotein cholesterol; ACR, albumin to creatinine ratio; eGFR, estimated glomerular filtration rate.

### 3.2 Associations between CDAI and risks of CKD

We analyzed the correlation between the CDAI score and the selected characteristics or laboratory variables. Spearman grade correlation analysis showed that PIR, education level, alcohol consumption, DM, DBP, Mn, Se, Zn, VA, VC, VE, TG, eGFR were positively correlated with CDAI score (Supplementary Table S3). In contrast, age, sex, marital status, CKD, BMI, WC, SBP, UCR, UA, HbA1c, SCR, SUA, HDL-C, ACR, GLU were negatively correlated with CDAI scores.

**Figure 2:**
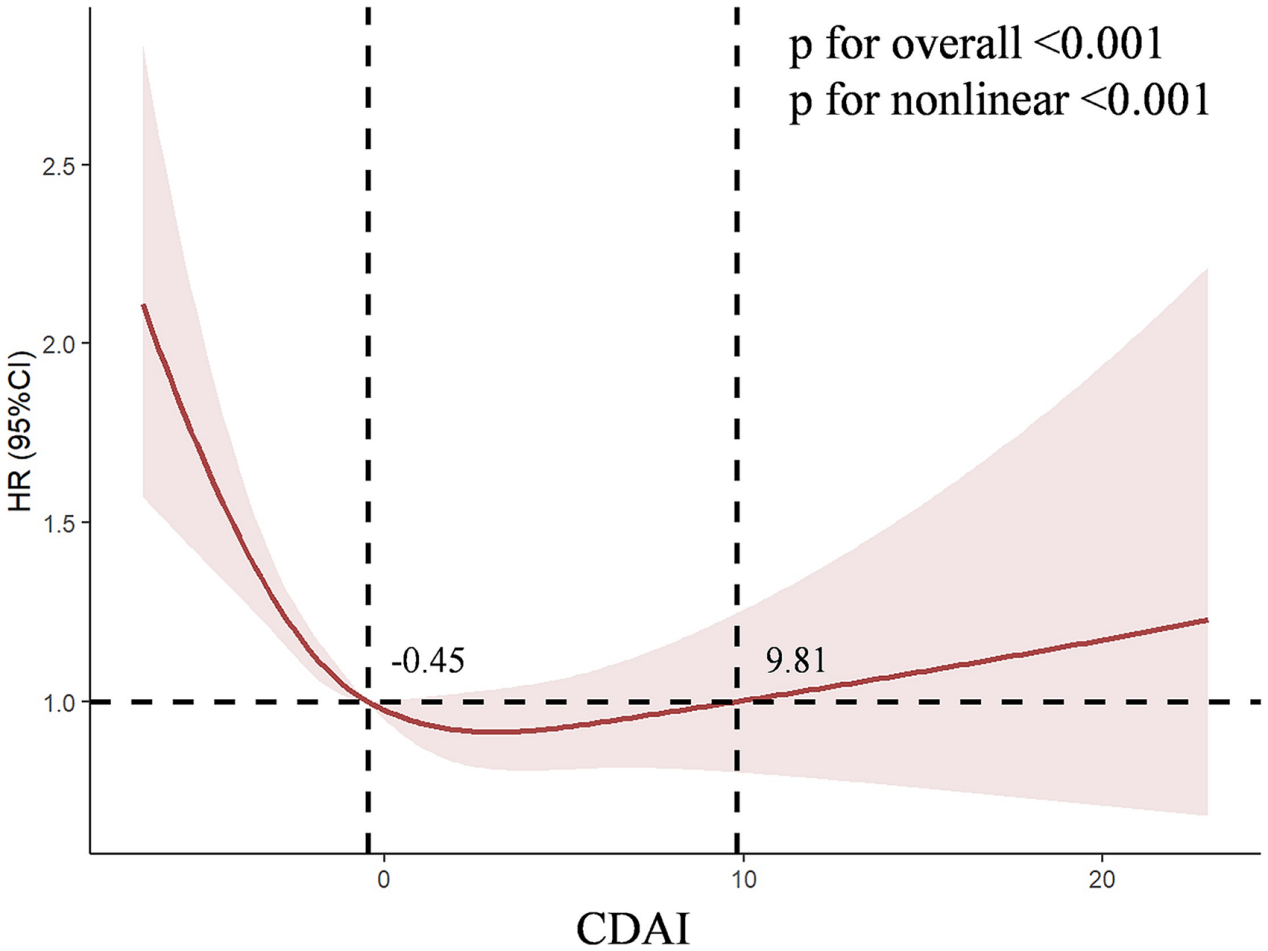
The dose–response relationships of CDAI with the prevalence of chronic kidney disease. The solid red line represents the smooth curve fit between variables. The shaded bands represent the 95% confidence intervals.

The logistic regression weighted model of CDAI and CKD was presented in Table 2. In the Model 3, CDAI was significantly negative correlated with CKD (OR = 0.802, 95%CI [0.753, 0.854], *p* < 0.001), which indicated that the prevalence of CKD was reduced for each additional unit rise in CDAI. After transforming the CDAI into quartiles, we found that individuals with the highest quartile of CDAI were 46.6% less likely to have CKD than those with the lowest quartile (OR = 0.534, 95% CI [0.437, 0.652], *p* < 0.001), and the trend test was also significant (*P* for trend < 0.001). This negative relationship remained stable in Q2 (−2.27 ~ −0.47) and Q3 (−0.47 ~ 1.17) of CDAI (OR = 0.742, 95% CI [0.624, 0.882], *p* < 0.001; OR = 0.585, 95% CI [0.486, 0.705], *p* < 0.001). According to the minimum principle of Akaike information criterion (AIC), restricted cubic spline (RCS) was used to flexibly model the association between CDAI and CKD. As shown in Figure 2, RCS indicated that the relationship between CDAI and CKD was non-linear (*P* for non-linear < 0.001).

**Table 2:** Associations between CDAI and CKD.

CDAI	Model 1		Model 2		Model 3	
	OR (95%CI)	*p*-value	OR (95%CI)	*p*-value	OR (95%CI)	*p*-value
As continuous	0.897(0.879,0.916)	<0.001	0.928(0.907,0.950)	<0.001	0.802(0.753,0.854)	<0.001
Inter-quartile						
Q1	Ref.		Ref.		Ref.	
Q2	0.704(0.604,0.819)	<0.001	0.731(0.615,0.868)	<0.001	0.742(0.624,0.882)	<0.001
Q3	0.503(0.427,0.594)	<0.001	0.566(0.470,0.681)	<0.001	0.585(0.486,0.705)	<0.001
Q4	0.393(0.329,0.468)	<0.001	0.512(0.420,0.624)	<0.001	0.534(0.437,0.652)	<0.001
*P* for trend	<0.001		<0.001		<0.001	

Model 1 was adjusted for none.

Model 2 was adjusted for age, gender, SBP and DBP.

Model 3 was adjusted for age, gender, SBP, DBP, PIR, drinking status, HbA1c.

**Figure 3:**
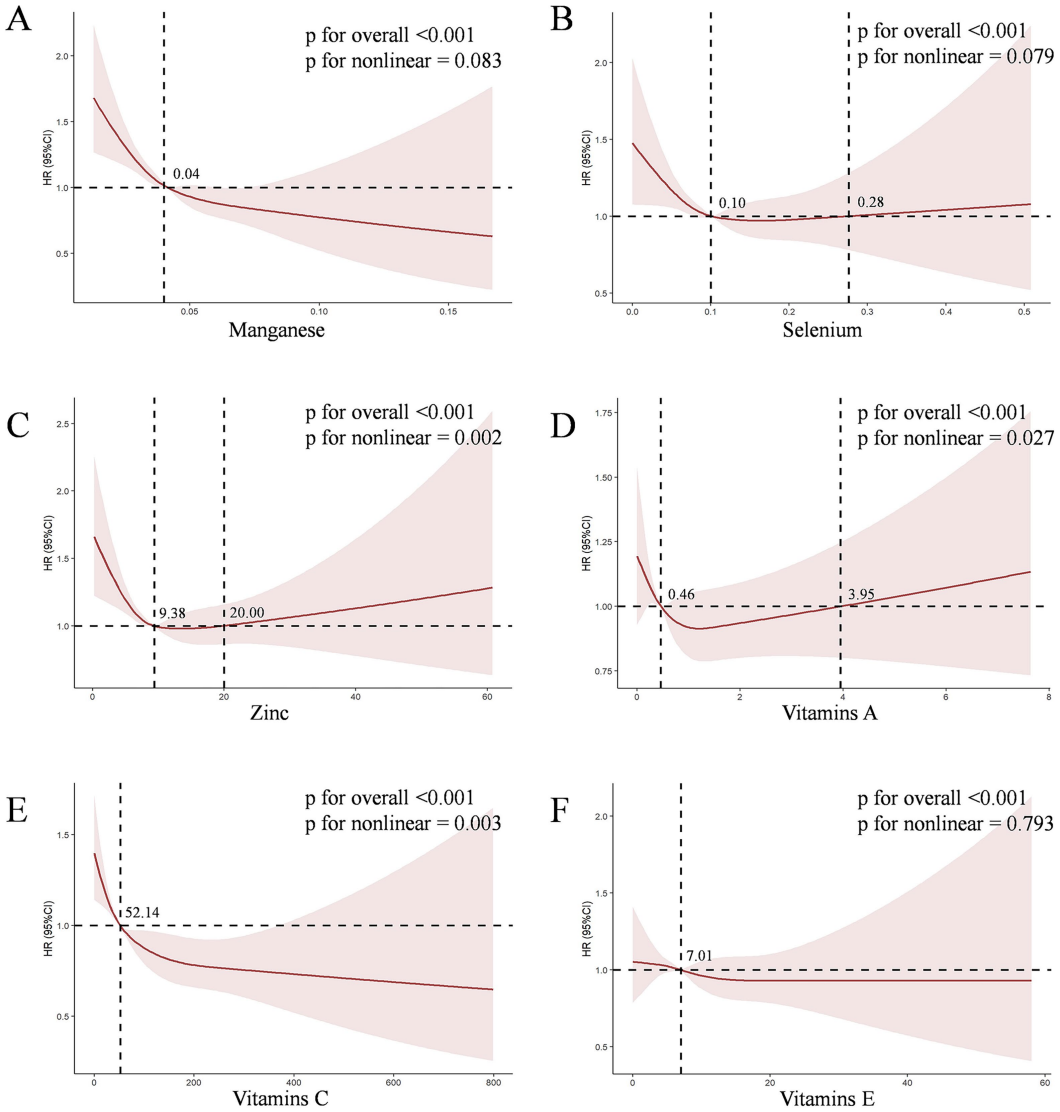
The dose–response relationships between six antioxidant components of CDAI and CKD. The solid red line represents the smooth curve fit between variables. The shaded bands represent the 95% confidence intervals. **(A)** The dose–response relationships of Manganese with CKD risk; **(B)** The dose–response relationships of Selenium with CKD risk; **(C)** The dose–response relationships of Zinc with CKD risk; **(D)** The dose–response relationships of Vitamins A with CKD risk; **(E)** The dose–response relationships of Vitamins C with CKD risk; **(F)** The dose–response relationships of Vitamins E with CKD risk.

We conducted a further analysis on the association between the six antioxidant components of CDAI and CKD. As shown in Supplementary Table S4, in Model 3, both manganese (HR = 0.000, 95% CI [0.000, 0.003], *p* < 0.001) and vitamin C (HR = 0.998, 95% CI [0.997, 0.999], *p* < 0.001) were independently associated with CKD. This HR value corresponds to a change per 1 mg/day increase in intake. To provide a more interpretable effect size, after standardizing manganese (where HR corresponds to a change per 0.064 mg/day increase in intake), the result for manganese was HR = 0.843, 95% CI [0.786–0.904], *p* < 0.001. Furthermore, after Bonferroni multiple comparison correction (with the significance threshold set at *p* < 0.0083), the associations for manganese and vitamin C remained highly significant (both *p*-values < 0.001), while the associations for the other components did not reach the corrected significance level. To further explore the nonlinear dose–response relationships between antioxidant components and CKD, we constructed the RCS for six antioxidant components (manganese, selenium, zinc, and vitamins A, C, E) and CKD in Model 3. The RCS showed a non-linear dose–response relationship between Zinc (*P* for non-linear = 0.002, V-shaped curve), vitamins A (*P* for non-linear = 0.027, V-shaped curve), vitamins C (*P* for non-linear = 0.003, L-shaped curve) levels and the prevalence of CKD, respectively (Figure 3).

### 3.3 Association among CDAI, FI and CKD

Spearman correlation analysis showed that there was a negative correlation between CDAI score and FI level (rs = − 0.107, *p* < 0.001), the higher the CDAI score, the lower FI level, but the correlation was weak (Supplementary Table S4).

Table 3 shows that continuous FI was positively associated with the occurrence of CKD. After adjusting for confounders, frail and pre-frail participants had significantly elevated risks of incident CKD than robust participants (HR = 1.799, 95% CI [1.448, 2.233]; HR = 1.616, 95% CI [1.291, 2.022], all *p* < 0.001). Further research has found that at the same level of CDAI, a higher FI is associated with a higher risk of developing CKD. After adjusting for confounding factors, participants who were frail and pre-frail had significantly higher risks of CKD events compared to healthy participants within the high CDAI group (HR = 1.965, 95% CI [1.208, 3.198], *p* = 0.007; HR = 1.919, 95% CI [1.164, 3.163], *p* = 0.011). This relationship was also observed in the low CDAI group (Frail HR = 2.294, 95% CI [1.558, 3.377]; Pre-frail HR = 1.967, 95% CI [1.316, 2.942], all p < 0.001). Trend tests indicated that there was a tendency for the risk of CKD events to increase with the overall Frailty Index (FI) (all trend *p*-values < 0.05) (Table 4).

**Table 3:** Associations between FI and the risks of CKD.

Frailty status	Model 1		Model 2		Model 3	
	HR (95%CI)	*p*-value	HR (95%CI)	*p*-value	HR (95%CI)	*p*-value
As continuous	67.372(53.095,85.488)	<0.001	9.134(6.904,12.084)	<0.001	3.743(2.800,5.006)	<0.001
Categories						
Robust	Ref.		Ref.		Ref.	
Pre-frail	4.688(3.761,5.842)	<0.001	1.990(1.591,2.488)	<0.001	1.616(1.291,2.022)	<0.001
Frail	11.228(9.150,13.777)	<0.001	2.693(2.177,3.330)	<0.001	1.799(1.448,2.233)	<0.001
*P* for trend	<0.001		<0.001		<0.001	

Model 1 was adjusted for none.

Model 2 was adjusted for age and gender.

Model 3 was adjusted for age, gender, HbA1c, Serum Creatinine, Serum Uric acid.

**Table 4:** Weighted association of FI with CKD in different levels of CDAI.

Variables	Model 1		Model 2		Model 3	
	HR [95%CI]	*p*-value	HR [95%CI]	*p*-value	HR [95%CI]	*p*-value
High CDAI						
Frailty status						
Continuous	89.651(49.213,163.317)	<0.001	6.393(3.188,12.823)	<0.001	5.597(2.748,11.401)	<0.001
Categories						
Robust	Ref		Ref		Ref	
Pre-frail	4.601(2.823,7.500)	<0.001	2.041(1.243,3.352)	0.005	1.919(1.164,3.163)	0.011
Frail	10.095(6.388,15.952)	<0.001	2.190(1.358,3.533)	0.001	1.965(1.208,3.198)	0.007
*P* for trend	<0.001		0.005		0.02	
Middle CDAI						
Frailty status						
Continuous	65.750(46.797,92.381)	<0.001	9.998(6.709,14.900)	<0.001	8.010(5.304,12.098)	<0.001
Categories						
Robust	Ref		Ref		Ref	
Pre-frail	4.382(3.195,6.010)	<0.001	1.871(1.357,2.579)	<0.001	1.679(1.216,2.320)	0.002
Frail	11.147(8.317,14.942)	<0.001	2.805(2.068,3.803)	<0.001	2.370(1.740,3.228)	<0.001
*P* for trend	<0.001		<0.001		<0.001	
Low CDAI						
Frailty status						
Continuous	47.216(31.308,71.205)	<0.001	8.942(5.484,14.581)	<0.001	7.683(4.627,12.757)	<0.001
Categories						
Robust	Ref		Ref		Ref	
Pre-frail	4.917(3.307,7.310)	<0.001	2.056(1.375,3.073)	<0.001	1.967(1.316,2.942)	<0.001
Frail	10.171(7.028,14.721)	<0.001	2.562(1.749,3.754)	<0.001	2.294(1.558,3.377)	<0.001
*P* for trend	<0.001		<0.001		<0.001	

For FI, 0 ≤ robust < 0.10, 0.10 ≤ pre-frail < 0.25, 0.25 ≤ frail < 1.

For CDAI, Low CDAI < −2.28, −2.28 ≤ Middle CDAI ≤ 1.72, High CDAI > 1.72.

Model 1 was adjusted for none.

Model 2 was adjusted for age, gender.

Model 3 was adjusted for age, gender, BMI, WC, SBP.

### 3.4 The mediation effects of FI in the relationships of CDAI with CKD risk

**Figure 4:**
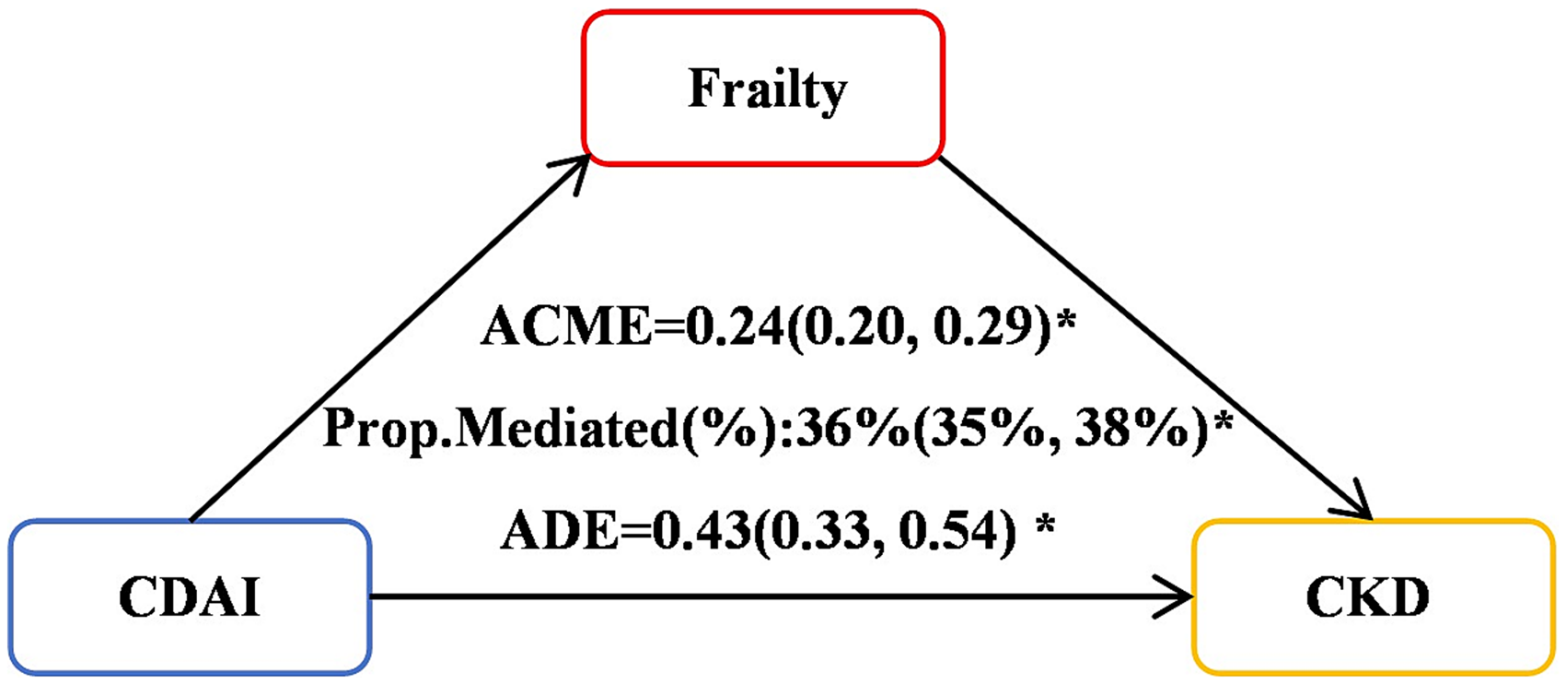
Mediation effects of FI in the associations of the six CDAI compounds with CKD risk. ACME, average causal mediation effects (indirect effect); ADE, average direct effects. *p < 0.05.

**Figure 5:**
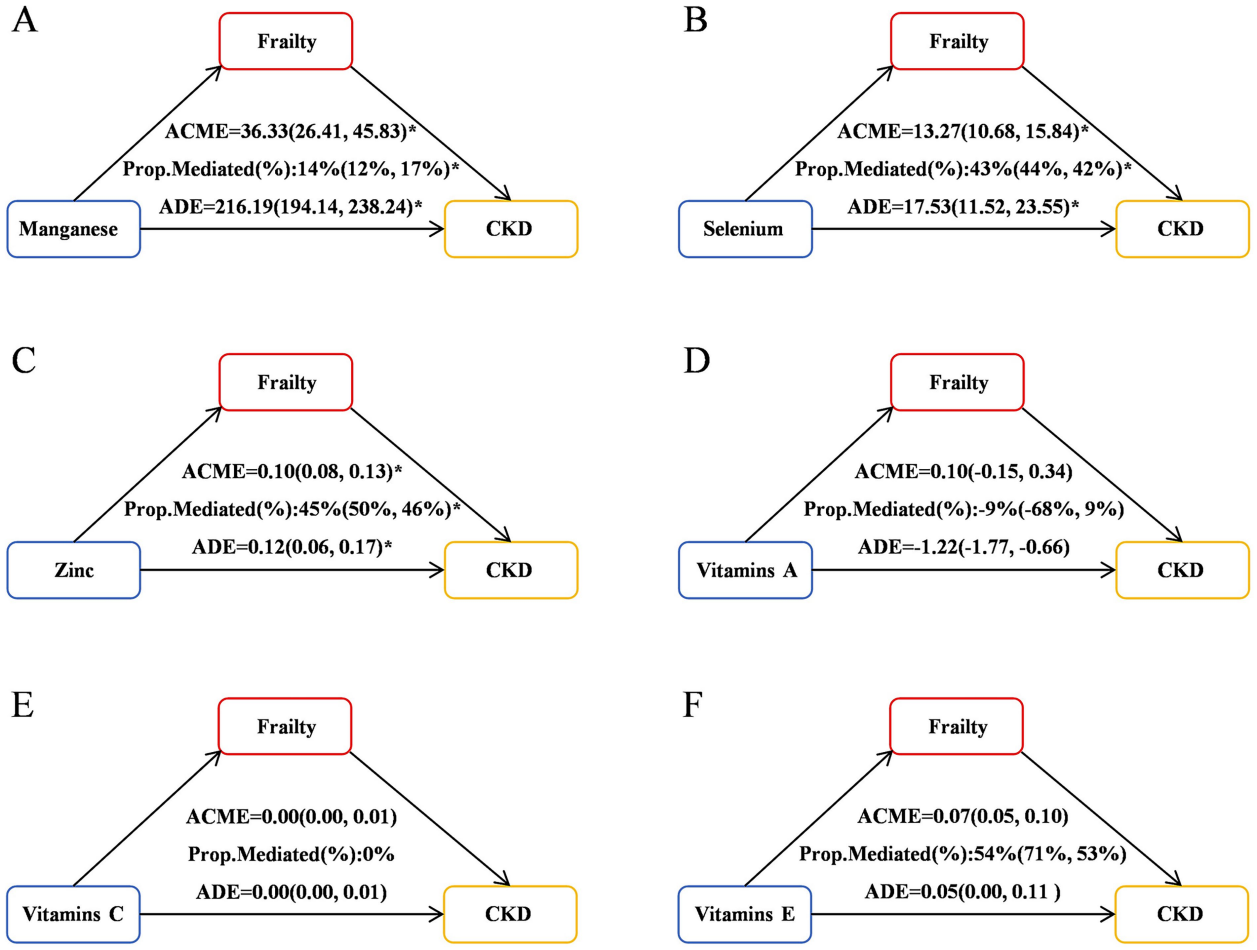
Mediation effects of FI in the associations of CDAI with CKD risk. ACME, average causal mediation effects (indirect effect); ADE, average direct effects. *p < 0.05. **(A)** Mediation effects of FI in the associations of Manganese with CKD risk; **(B)** Mediation effects of FI in the associations of Selenium with CKD risk; **(C)** Mediation effects of FI in the associations of Zinc with CKD risk; **(D)** Mediation effects of FI in the associations of Vitamins A with CKD risk; **(E)** Mediation effects of FI in the associations of Vitamins C with CKD risk; **(F)** Mediation effects of FI in the associations of Vitamins E with CKD risk.

Significant mediation effects of FI were observed in the relationships between CDAI and CKD risk. The mediated proportion (%) of FI in the relationships of CDAI with CKD risk were 36% (95%CI: 35, 38%) (Figure 4). We further analyzed and observed significant mediation effects of FI in the relationship between the six CDAI compounds and CKD risk. The mediated proportion (%) of FI in the relationships of Manganese, Selenium and Zinc with CKD risk were 14% (95%CI: 12, 17%), 43% (95%CI:44, 42%) and 45% (95%CI:50, 46%), respectively (Figure 5).

### 3.5 Survival patterns of CKD population in different levels of CDAI

**Figure 6:**
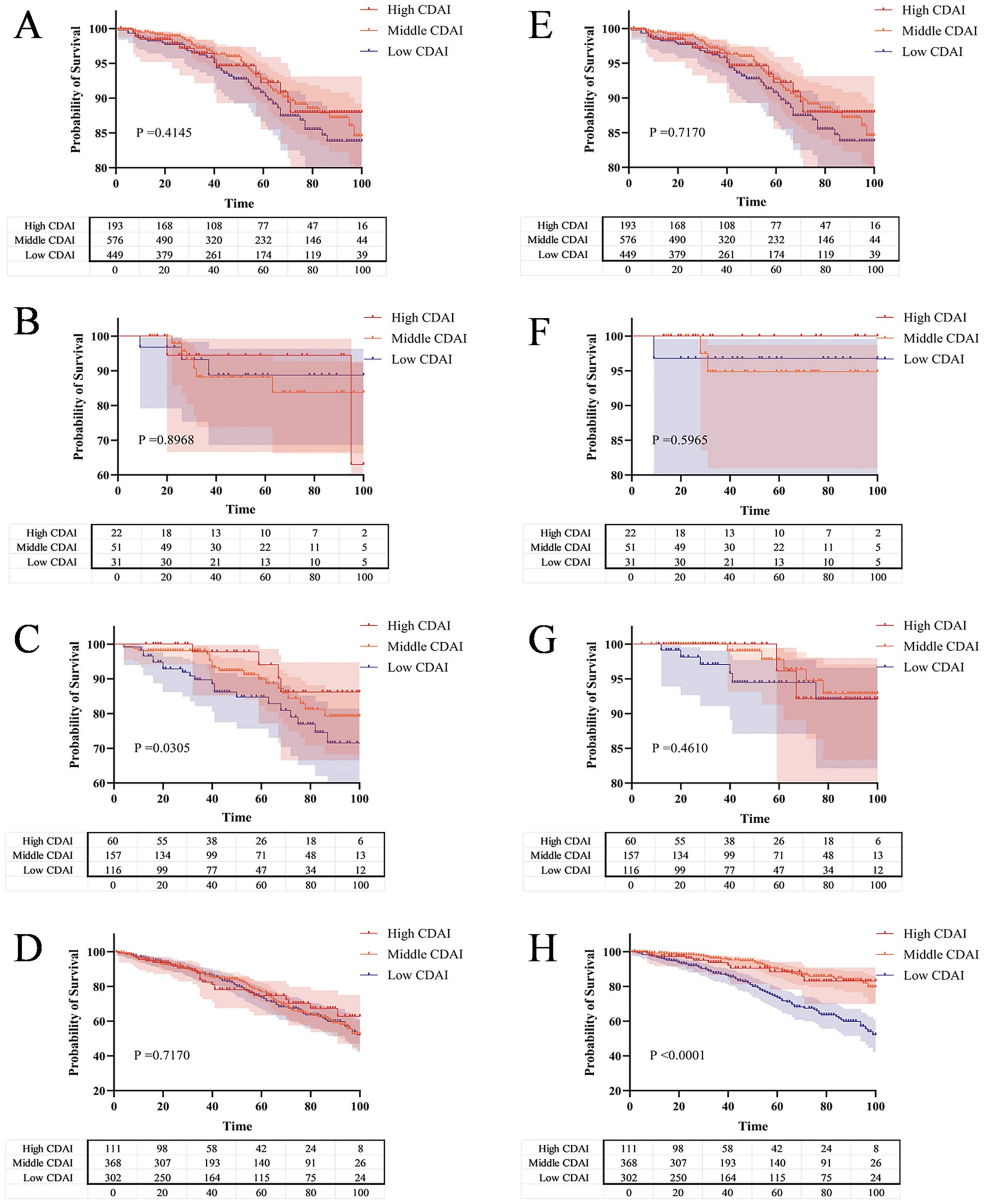
The Kaplan–Meier curve shows the all-cause and cardiovascular mortality probabilities of CKD patients with different CDAI levels under the same FI level. **(A)** All-cause mortality risk across different levels of CDAI; **(B)** All-cause mortality risk across different levels of CDAI in the robust group; **(C)** All-cause mortality risk across different levels of CDAI in the pre-frail group; **(D)** All-cause mortality risk across different levels of CDAI in the frail group; **(E)** Cardiovascular mortality risk across different levels of CDAI; **(F)** Cardiovascular mortality risk across different levels of CDAI in the robust group; **(G)** Cardiovascular mortality risk across different levels of CDAI in the pre-frail group; **(H)** Cardiovascular mortality risk across different levels of CDAI in the frail group.

The KM curves reveal that within the same level of FI, patients with CKD who have lower CDAI exhibit higher all-cause mortality and cardiovascular mortality rates. Meanwhile, when the frailty status of CKD patients was not distinguished, different CDAI levels had no significant effect on all-cause mortality or cardiovascular mortality in CKD patient (*p* = 0.4145 and *p* = 0.7170) (Figures 6A, E). For all-cause mortality, pre-frail CKD patients with lower CDAI had lower survival rates (*p* = 0.0305) (Figure 6C), while this phenomenon was not observed in robust or frail CKD patients (*p* = 0.8968 and *p* = 0.7170) (Figures 6B, D). Regarding cardiovascular mortality, frail CKD patients with lower CDAI had lower survival rates (*p* < 0.0001) (Figure 6H), which was not the case for robust or pre-frail CKD patients (*p* = 0.5965 and *p* = 0.4610) (Figures 6F, G).

We conducted a further analysis on the association between the six antioxidant components of CDAI and Mortality. As shown in Supplementary Table S5, after full adjusted, vitamins A (HR = 1.266, 95% CI [1.072, 1.495], *p* = 0.005) were positively associated with Cardiovascular Mortality. However, a negative correlation was observed between the vitamins E and cardiovascular mortality (Full adjusted: HR = 0.934, 95%CI [0.883, 0.989], *p* = 0.019).

### 3.6 Sensitivity analyses

When comparing the weighted associations of FI with CKD across different levels of CDAI in NHANES participants between 2011 and 2014 and 2015–2018, consistent results were observed in both cycles (Supplementary Table S7). At the same CDAI level, the progression of frailty status still increased the risk of CKD occurrence, while the recovery of frailty status reduced the risk of CKD occurrence. After further excluding extreme values of CDAI, the association between changes in frailty status and CKD was also consistent (Supplementary Table S8). Our in-depth subgroup analysis showed that the strong association between FI and CKD was prevalent across subgroups with different ages, genders, BMIs, and marital statuses. What’s more, the results were highly consistent (all HR > 1, mostly *p* < 0.05). At the same CDAI level, the HR value was significantly higher in people aged < 60 years than in those aged > 60 years. Especially at the low CDAI level, the gap in hazard ratios reached 77.69%. For gender, BMI, and marital status, although the HRs fluctuated across different subgroups, the gaps were not significant. This strongly supports the robustness and generality of our main conclusion, indicating that the role of frailty as a risk factor for CKD is stable across different population characteristics (Supplementary Table S9).

## 4 Discussion

In this multi-cycle NHANES study targeting adults in the United States, we found a negative correlation between the CDAI and the occurrence and progression of CKD, and identified frailty as a mediator in this relationship. Firstly, we observed a significant negative correlation between CDAI and CKD across various logistic regression models. RCS analysis revealed a non-linear association between CDAI and CKD (*P* for non-linear < 0.001). Among the six antioxidant components of CDAI, manganese and vitamin C were independently associated with the presence of CKD. RCS analysis further demonstrated non-linear dose–response relationships between zinc, vitamins A and C levels, and the prevalence of CKD. Secondly, our research results indicate a weak negative correlation between the level of FI and the CDAI score, while there is a positive correlation between FI and the occurrence of CKD. What’s more, mediation analysis revealed significant mediation effects of FI in the relationships between CDAI and CKD risk, with a mediated proportion of 36% (95% CI: 35, 38%). Similarly, FI mediated the relationships between manganese, selenium, zinc, and CKD risk, with the mediated proportion ranging from 14 to 45%, respectively. Finally, survival analysis using KM curves revealed that, among patients with CKD grouped into frail and pre-frail categories, those with lower CDAI levels had significantly reduced overall and cardiovascular-specific survival probabilities compared to those with higher CDAI levels. Further analysis revealed that vitamin A was positively associated with cardiovascular mortality, while vitamin E was negatively associated with it.

Although existing research on the relationship between CDAI and CKD is still relatively rare, the use of dietary antioxidant properties to intervene CKD has been a hot topic of research today. Multiple studies have shown that the exogenous intake of antioxidants prevents inflammation, atherosclerosis, insulin resistance, and oxidative stress in CKD and dialysis patients (25–27). A study indicates that a higher total dietary antioxidant capacity is associated with a reduced incidence of CKD (28). In line with previous studies, our results also confirmed a negative correlation between CDAI and CKD. Further analysis indicates that six components of the CDAI are negatively correlated with CKD. A study found that selenium and zinc intake in the diets of Chinese adults was negatively correlated with CKD (29), while two studies conducted in Canada and the Czech Republic showed no significant relationship (30, 31). Therefore, this correlation may be influenced by different ethnicities.

A key contribution of the present study is the novel identification of the significant mediating role of the FI in the association between CDAI and CKD risk. Previous studies have explored the impact of CDAI on FI or the influence of FI on CKD risk, but they have treated FI as an independent risk factor for adverse outcomes or specific adverse outcomes (such as mortality) (32). For example, a cross-sectional study from NHANES 2003–2018 examine the relationships between the CDAI and frailty and the underlying mechanisms involved (10). They found that frailty was negatively correlated with CDAI scores, which may be partially mediated by oxidative stress. In this study, we conducted directed acyclic graph (DAG) analysis to explore the relationships among the FI, CDAI and CKD, and proposed the hypothesis that FI may play a crucial mediating role in the association between low CDAI and CKD risk. Our study observed a significant mediating effect of FI on the correlation between CDAI and CKD risk, with a mediation proportion of 36% (95% CI: 35, 38%). Furthermore, FI also exerted a significant mediating role in the relationships between the components of CDAI (manganese, selenium, zinc) and CKD risk, with mediation proportions of 14, 43, and 45%, respectively.

The mechanism by which FI mediates the relationship between CDAI and CKD may involve multiple interconnected biological pathways, including oxidative stress, chronic inflammation, and gut microbiota dysbiosis. Firstly, antioxidant nutrients in CDAI (such as vitamins C and E, manganese, selenium, etc.) alleviate oxidative damage by neutralizing ROS, activating the Nrf2 antioxidant pathway, and other mechanisms. In contrast, frailty is often accompanied by reduced antioxidant capacity and elevated oxidative stress levels (33, 34). Such elevated oxidative stress can directly damage glomerular and tubular cells, accelerating the progression of CKD. Therefore, a lower CDAI may fail to effectively suppress oxidative stress, exacerbating frailty and thereby promoting CKD. Secondly, frailty is considered a chronic inflammatory state. Our study and multiple literature sources indicate that a higher CDAI is associated with lower systemic inflammation levels [e.g., C-reactive protein (CRP), interleukin-6 (IL-6)] (35). Conversely, frail individuals often exhibit elevated levels of pro-inflammatory cytokines. Chronic inflammation is a core mechanism in the onset and progression of CKD, as it can impair renal function by promoting renal fibrosis and vascular sclerosis (36). Thus, CDAI may alleviate frailty by suppressing inflammatory pathways, indirectly reducing the risk of CKD. Additionally, intestinal dysbiosis, impaired intestinal barrier function, and reduced renal clearance of bacterial metabolites may all increase the risk of complications in patients with CKD (37). In the later stages of CKD, the accumulation of waste products and inadequate fiber intake can alter the gut microbiome, leading to dysbiosis (38). Dysbiosis can increase levels of toxic compounds such as trimethylamine N-oxide (TMAO), indoxyl sulfate, and p-cresyl sulfate, contributing to increased cardiovascular risk in CKD patients (38–40). By implementing dietary adjustments, the healthy balance of the gut microbiome in patients with advanced CKD can be partially restored and levels of toxic metabolites reduced (41). Therefore, early intervention through dietary antioxidant intake may delay the progression of CKD and significantly reduce cardiovascular mortality in patients with advanced CKD. In summary, FI likely serves as a comprehensive physiological indicator reflecting multiple physiological disruptions triggered by antioxidant deficiency—elevated oxidative stress and chronic inflammation, and gut ecological imbalance. These disruptions collectively form important mediating pathways through which CDAI influences CKD risk.

Although numerous studies have highlighted the potential value of CDAI in predicting the risk of CKD onset, research exploring its prognostic role in populations with established CKD remains relatively limited. To our knowledge, this study is the first to determine the prognostic value of CDAI in a US CKD population across different levels of FI. Our findings indicate that, at the same FI level, participants with a lower CDAI exhibited significantly elevated risks of both all-cause mortality and cardiovascular disease mortality. A noteworthy observation was the distinct pattern of associations: for all-cause mortality, pre-frail CKD patients benefited from dietary antioxidant intake, whereas frail patients did not; conversely, for cardiovascular mortality, pre-frail CKD patients did not benefit from dietary antioxidant intake, but frail patients did.

This may be explained by the fact that in the pre-frail state, patients retain a greater degree of physiological reserve. Here, the primary threat is likely the cumulative burden of oxidative stress driving multi-organ functional decline. A lower CDAI, indicating poorer antioxidant intake, fails to mitigate this burden, leading to a higher risk of all-cause mortality (9). The protective effect of CDAI against cardiovascular events probably still exists but may be diluted within the higher overall risk of death from various causes in this group. Upon transitioning to the frail state, patients exhibit a profound loss of physiological reserve and are often in a state of severe chronic inflammation and catabolism (42). In this context, the body’s ability to utilize nutrients (such as antioxidants) may be significantly impaired, making it difficult for dietary differences to manifest as a clear survival signal in all-cause mortality. However, the cardiovascular system might remain particularly sensitive to oxidative insult even in advanced frailty (9, 43). The significantly elevated risk of cardiovascular mortality associated with lower CDAI in frail patients suggests that micronutrient status (high CDAI level) remains critically important for maintaining cardiomyocyte viability and preventing fatal arrhythmias or heart failure until the very end stages of disease (44). In summary, different frailty statuses indicate a shift in the role of the CDAI: from a generalized protective effect of antioxidants against multi-organ functional decline in pre-frail individuals, to a more focused, critical role in preserving vital cardiac function in the most severely compromised frail patients. Therefore, early intervention through dietary antioxidant intake may delay the progression of CKD and significantly reduce cardiovascular mortality in patients with advanced CKD.

We further analyzed the relationships between the six antioxidant components of CDAI and mortality, founding that vitamin A exhibited a counterintuitive positive association with cardiovascular mortality (HR = 1.266, *p* = 0.005). This may be related to its complex metabolic processes and potential toxicity at high doses, especially in the context of impaired renal function. The RCS analysis of vitamin A (Figure 3D) revealed a significant non-linear “V-shaped” association with CKD prevalence (*P* for non-linear = 0.027). This curve suggests that there is a range of vitamin A intake that may exert protective or neutral effects, and beyond this range, the risk may increase. Relevant literature also confirms the multifaceted nature of vitamin A’s effects. A study by Tschuck et al. (43) found that vitamin A can act both as a direct radical-trapping antioxidant (e.g., retinol, retinal) and as a regulator that activates transcriptional programs inhibiting cell death (such as ferroptosis) via retinoic acid receptors (e.g., all-trans retinoic acid). However, in the impaired internal environment of uremia, this signaling transduction may become dysregulated. Instead of activating protective anti-ferroptotic genes, vitamin A may drive pro-inflammatory or pro-fibrotic pathways in the cardiovascular system. A study by Zinder et al. (45) even explicitly stated: “When considering supplementation, the potential benefits must be weighed against the risk of harm. Vitamin A toxicity can be critical and even result in death.” This dose-dependent complex effect stands in sharp contrast to the relatively direct protective effect of vitamin E. Vitamin E showed a stable linear negative association with cardiovascular mortality (HR = 0.934, *p* = 0.019). As a pure lipid-soluble antioxidant, its primary role is to scavenge free radicals in cell membranes—a mechanism that generally exerts universal protective effects without the risk of receptor-mediated toxicity. Manganese and selenium also exhibit protective effects at dietary intake levels (44, 46). However, as elaborated in the literature on manganese neurotoxicity by Zhang et al. (46), excessive exposure to these elements may also cause harm, which underscores the “dual-effect” characteristic of many micronutrients. Therefore, we need to abandon the misconception that “more is better” when it comes to antioxidants. Nutritional guidance should focus on obtaining a balanced combination of antioxidants primarily from food sources (such as colorful vegetables, nuts, and seeds), with particular emphasis on nutrients with a wider therapeutic window, such as vitamin E and selenium. Meanwhile, caution should be exercised regarding high-dose vitamin A supplementation in patients with advanced CKD.

The nutritional management of patients with CKD aims to slow disease progression, control symptoms, and improve quality of life. Since the kidneys play a key role in regulating body fluids, electrolytes, and metabolic wastes, impaired renal function exerts a significant impact on nutritional status. CDAI is an indicator that measures the antioxidant capacity of the diet. Evidence suggests that an antioxidant-rich diet can prevent CKD, yet it does not provide effective intervention measures (28). Meanwhile, CKD is closely associated with malnutrition and sarcopenia, necessitating nutritional strategies to prevent muscle loss and sarcopenia (47, 48). Rehabilitation nutrition is an intervention strategy for improving nutritional status and physical function, but its application in CKD patients has not been fully elucidated (48). Nutrition is the foundation of sustaining life and health; a scientific and reasonable diet can not only meet the body’s requirements for various nutrients but also is crucial for disease prevention and health maintenance. Based on relevant reports and our research, we recommend that everyone consume at least 300 g of fresh fruits and vegetables daily (with dark-colored vegetables accounting for more than half). It is recommended that adults consume 50–100 g of whole grains (such as brown rice, oats, quinoa, and whole wheat) per day, with whole grains included in at least one of the three daily meals (whole grains are important sources of dietary fiber, B vitamins, and minerals) (49). Fat-rich fish (such as salmon, eel, and mackerel) are good sources of vitamin D and vitamin A. Shellfish (such as clams and oysters) are good sources of iron, zinc, iodine, and other minerals. It is recommended to consume aquatic products such as fish, shrimp, and shellfish 1–2 times a week, with an intake of approximately 300–500 grams for adults. Children, pregnant women, lactating women, and the elderly are encouraged to appropriately increase their intake of aquatic products. However, considering that populations of different ages, genders, and physiological conditions have different physiological needs, we advocate more for formulating personalized dietary plans based on individual circumstances (50).

An important strength of this study is the in-depth analysis of the relationships among the CDAI, FI, CKD and mortality, as well as the further exploration of each antioxidant component in CDAI. This provides in-depth insights into the association between diet and CKD. Moreover, this study innovatively identified the significant mediating role of FI in the relationship between CDAI and the risk of CKD, offering a new perspective for understanding the pathogenesis of CKD and nutritional interventions. Additionally, we used a large and representative sample along with multiple statistical methods to ensure the reliability and accuracy of the research results. However, our study also has some limitations. Firstly, due to its cross-sectional design, we cannot determine the causal relationships among CDAI, FI, and CKD. Secondly, although multiple variables such as age, gender, and various health-related factors were adjusted, there may still be unmeasured or difficult-to-measure confounding factors that could affect the research results. Finally, although the construction of the FI in this study was limited to 9 items due to data availability constraints in the NHANES dataset, the index still demonstrated significant validity in predicting CKD risk and mortality, supporting its utility in the context of this research. It should be acknowledged that a more comprehensive FI comprising 30–40 items, as suggested by Searle et al. (20), might capture more nuanced gradients of frailty. Therefore, future studies with access to richer variable datasets could further refine the assessment of frailty in CKD populations.

## 5 Conclusion

In summary, this cross-sectional study based on four cycles (2011–2018) of NHANES found a negative correlation between the CDAI and CKD among American adults after adjusting for potential confounding factors, and emphasized the mediating role of frailty in this relationship. This study provides new insights into exploring the potential benefits of dietary adjustments in preventing the progression of CKD, especially in vulnerable populations. In the future, there is an urgent need for more randomized controlled trials or cohort studies to confirm this finding and to provide more accurate and effective prevention and treatment options for integrating nutrition and geriatric care.

## Data Availability

Publicly available datasets were analyzed in this study. This data can be found: data are available from the National Health and Nutrition Examination Survey (NHANES) repository, https://www.cdc.gov/nchs/nhanes/index.htm. No specific accession number as data are publicly accessible via the official website.

## Footnotes

^1^ https://www.cdc.gov/nchs/nhanes/
