# Supplementary material for: Comprehensive dietary antioxidant index and chronic kidney disease: mediating role of frailty and its impact on mortality outcomes in adults
Source: Front Nutr. 2025 Oct 14;12:1679774. doi: 10.3389/fnut.2025.1679774 (PMC12558786; doi:10.3389/fnut.2025.1679774)
Supplement: Supplementary file 1 [file Table_1.docx]

Supplementary Material

**Table S1** The 9 items used to construct the frailty index

| No | Description of the item | Cut-off value |
| --- | --- | --- |
|  | NHANES |  |
| 1 | Self-reported physician diagnosed hypertension | Yes = 1, No = 0 |
| 2 | Self-reported physician diagnosed cancer | Yes = 1, No = 0 |
| 3 | Self-reported physician diagnosed arthritis | Yes = 1, No = 0 |
| 4 | Self-reported physician diagnosed coronary heart disease | Yes = 1, No = 0 |
| 5 | Self-reported general health status | Yes = 1, No = 0 |
| 6 | Self-reported physician diagnosed celiac disease | Yes = 1, No = 0 |
| 7 | Self-reported general gallstones | Yes = 1, No = 0 |
| 8 | days of poor physical health per month | good<15, poor≥15 |
| 9 | days of poor mental health per month | good<15, poor≥15 |

NHANES, National Health and Nutrition Examination Survey.

**Table S2.** The missing number and rate of covariates of changes in CDAI score

| **Variables** | **NHANES** | | | |
| --- | --- | --- | --- | --- |
|  | **CDAI≤-2.28(n=2966)** | **-2.28<CDAI<1.72(n=5965)** | **CDAI≥1.72(n=2973)** | **All(n=11904)** |
| **Clinical characteristics** | | | | |
| **Age** | 0(0.00) | 0(0.00) | 0(0.00) | 0(0.00) |
| **Sex** | 0(0.00) | 0(0.00) | 0(0.00) | 0(0.00) |
| **PIR** | 318(10.70) | 504(8.40) | 221(7.40) | 1043(8.80) |
| **Education** | 0(0.00) | 0(0.00) | 0(0.00) | 0(0.00) |
| **Marriage** | 0(0.00) | 0(0.00) | 0(0.00) | 0(0.00) |
| **Drinking status** | 0(0.00) | 0(0.00) | 0(0.00) | 0(0.00) |
| **DM** | 0(0.00) | 0(0.00) | 0(0.00) | 0(0.00) |
| **CKD** | 0(0.00) | 0(0.00) | 0(0.00) | 0(0.00) |
| **BMI** | 24(0.80) | 28(0.50) | 25(0.80) | 77(0.60) |
| **WC** | 104(3.50) | 122(2.00) | 64(2.20) | 290(2.40) |
| **SBP** | 215(7.20) | 393(6.60) | 186(6.30) | 794(6.70) |
| **DBP** | 215(7.20) | 393(6.60) | 186(6.30) | 794(6.70) |
| **Laboratory data** | | | | |
| **Mn** | 0(0.00) | 0(0.00) | 0(0.00) | 0(0.00) |
| **Se** | 0(0.00) | 0(0.00) | 0(0.00) | 0(0.00) |
| **Zn** | 0(0.00) | 0(0.00) | 0(0.00) | 0(0.00) |
| **VA** | 0(0.00) | 0(0.00) | 0(0.00) | 0(0.00) |
| **VC** | 0(0.00) | 0(0.00) | 0(0.00) | 0(0.00) |
| **VE** | 0(0.00) | 0(0.00) | 0(0.00) | 0(0.00) |
| **UCR** | 0(0.00) | 0(0.00) | 0(0.00) | 0(0.00) |
| **UA** | 0(0.00) | 0(0.00) | 0(0.00) | 0(0.00) |
| **HbA_1c_** | 4(0.10) | 5(0.10) | 2(0.10) | 11(0.10) |
| **SCR** | 0(0.00) | 0(0.00) | 0(0.00) | 0(0.00) |
| **SUA** | 2(0.10) | 3(0.10) | 1(0.10) | 6(0.10) |
| **TG** | 3(0.10) | 6(0.10) | 1(0.10) | 10(0.10) |
| **HDL-C** | 2(0.10) | 1(0.10) | 1(0.10) | 4(0.00) |
| **ACR** | 0(0.00) | 0(0.00) | 0(0.00) | 0(0.00) |
| **eGFR** | 0(0.00) | 0(0.00) | 0(0.00) | 0(0.00) |

NHANES, National Health and Nutrition Examination Survey; PIR, Price-to-Income Ratio; DM, Diabetes Mellitus; CKD, Chronic kidney disease; BMI, Body mass index; WC, Waist Circumference; SBP, Systolic blood pressure; DBP, Diastolic blood pressure; VA, Vitamin A; VC, Vitamin C; VE, Vitamin E; UCR, Urine Creatinine; UA, Urine Albumin; HbA1c, Glycated Hemoglobin A1c; SCR, Serum Creatinine; SUA, Serum Uric acid; TG, Triglyceride; HDL-C, High Lipoprotein Cholesterol; ACR, Albumin to Creatinine Ratio; eGFR, estimated Glomerular Filtration Rate

**Table S3.** Correlation between CDAI score and baseline data

| **Variables** | **CDAI** | |
| --- | --- | --- |
|  | **Correlation** | **P-value** |
| Clinical characteristics | | |
| Age | -0.1 | <0.001 |
| Sex | -0.14 | <0.001 |
| PIR | 0.11 | <0.001 |
| Education | 0.12 | <0.001 |
| Marriage | -0.11 | 0.089 |
| Drinking status | 0.02 | 0.009 |
| DM | 0.07 | <0.001 |
| CKD | -0.1 | <0.001 |
| BMI | -0.03 | 0.007 |
| WC | -0.02 | 0.072 |
| SBP | -0.07 | <0.001 |
| DBP | 0.03 | 0.006 |
| Laboratory data | | |
| Mn | 0.27 | <0.001 |
| Se | 0.61 | 0 |
| Zn | 0.59 | 0 |
| VA | 0.43 | 0 |
| VC | 0.44 | 0 |
| VE | 0.56 | 0 |
| UCR | -0.05 | <0.001 |
| UA | -0.04 | <0.001 |
| HbA1c | -0.05 | <0.001 |
| SCR | -0.04 | <0.001 |
| SUA | -0.01 | 0.419 |
| TG | 0.02 | 0.015 |
| HDL-C | -0.03 | 0.003 |
| ACR | -0.028 | 0.002 |
| eGFR | 0.111 | <0.001 |

NHANES, National Health and Nutrition Examination Survey; PIR, Price-to-Income Ratio; DM, Diabetes Mellitus; CKD, Chronic kidney disease; BMI, Body mass index; WC, Waist Circumference; SBP, Systolic blood pressure; DBP, Diastolic blood pressure; VA, Vitamin A; VC, Vitamin C; VE, Vitamin E; UCR, Urine Creatinine; UA, Urine Albumin; HbA1c, Glycated Hemoglobin A1c; SCR, Serum Creatinine; SUA, Serum Uric acid; TG, Triglyceride; HDL-C, High Lipoprotein Cholesterol; ACR, Albumin to Creatinine Ratio; eGFR, estimated Glomerular Filtration Rate.

**Table S4.** Association of six components of CDAI and CKD.

| **Components** | **Model 1** | | **Model 2** | | **Model 3** | |
| --- | --- | --- | --- | --- | --- | --- |
|  | **HR (95%CI)** | **p-value** | **HR (95%CI)** | **p-value** | **HR (95%CI)** | **p-value** |
| Manganese | 0.000(0.000,0.000) | <0.001 | 0.000(0.000,0.001) | <0.001 | 0.000(0.000,0.003) | <0.001 |
| Manganese^^^ | 0.668(0.620,0.719) | <0.001 | 0.821(0.765,0.882) | <0.001 | 0.843(0.786,0.904) | <0.001^*^ |
| Selenium | 0.005(0.002,0.015) | <0.001 | 0.307(0.102,0.922) | 0.035 | 0.316(0.105,0.948) | 0.04 |
| Zinc | 0.962(0.952,0.972) | <0.001 | 0.987(0.978,0.997) | 0.014 | 0.990(0.980,1.000) | 0.048 |
| Vitamins A | 1.030(0.968,1.096) | 0.352 | 0.951(0.872,1.037) | 0.252 | 0.979(0.905,1.059) | 0.594 |
| Vitamins C | 0.998(0.998,0.999) | <0.001 | 0.998(0.997,0.999) | <0.001 | 0.998(0.997,0.999) | <0.001^*^ |
| Vitamins E | 0.972(0.962,0.983) | <0.001 | 0.990(0.979,1.000) | 0.052 | 0.994(0.984,1.004) | 0.265 |

^ The standardized HR represents the risk change for each 1 standard deviation (0.064 mg/day) increase in intake.

* For those with still significant P-values after Bonferroni correction (P < 0.0083).

Model 1 was adjusted for none.

Model 2 was adjusted for age and marriage.

Model 3 was adjusted for age, marriage, drinking status, diabetes, urine albumin.

**Table S5.** Correlation between CDAI score and FI level by Spearman analysis.

| **Variable** | **Correlation Coefficient** | ***P*-value** |
| --- | --- | --- |
| FI level | -0.107** | <0.001 |

** Correlation is significant at the 0.01 level(2-tailed).

**Table S6.** Association of six components of CDAI and Mortality.

| **Components** | **All-cause Mortality** | | **Cardiovascular Mortality** | |
| --- | --- | --- | --- | --- |
|  | **HR(95%CI)** | **p-value** | **HR(95%CI)** | **p-value** |
| Manganese | 0.002(0.000,11.273) | 0.157 | 0.000(0.000,888.721) | 0.289 |
| Selenium | 0.227(0.010,4.928) | 0.345 | 2.578(0.022,295.726) | 0.696 |
| Zinc | 1.020(0.997,1.043) | 0.083 | 1.026(0.993,1.061) | 0.121 |
| Vitamins A | 1.074(0.914,1.263) | 0.383 | 1.266(1.072,1.495) | 0.005* |
| Vitamins C | 0.999(0.998,1.001) | 0.566 | 1.000(0.997,1.003) | 0.992 |
| Vitamins E | 0.972(0.941,1.004) | 0.084 | 0.934(0.883,0.989) | 0.019* |

All-cause Mortality was adjusted for age, gender, PIR, education, BMI, WC, SBP, DBP, Serum Creatinine. * P < 0.05. Cardiovascular Mortality was adjusted for age, gender, PIR, BMI, WC, SBP, DBP, HbA1c, Serum Creatinine, Serum Uric acid, eGFR. * P < 0.05.

**Table S7.** Association of FI with CKD across different levels of CDAI in NHANES participants between 2011-2014 and 2015-2018.

| **Variables** | **NHANES (2011-2014)** | | **NHANES (2015-2018)** | |
| --- | --- | --- | --- | --- |
|  | **HR [95%CI]** | ***p*-value** | **HR [95%CI]** | ***p*-value** |
| **High CDAI** | | | | |
| Frailty status | | | | |
| Continuous | 1.448(1.061,1.975) | <0001 | 1.246(0.940,1.652) | 0.127 |
| Categories | | | | |
| Robust | Ref |  | Ref |  |
| Pre-frail | 2.354(1.092,5.078) | 0.029 | 2.065(1.066,3.999) | 0.032 |
| Frail | 2.604(1.239,5.475) | 0.012 | 1.880(0.983,3.593) | 0.056 |
| *P* for trend | 0.040 | | 0.092 | |
| **Middle CDAI** | | | | |
| Frailty status | | | | |
| Continuous | 1.533(1.271,1.848) | <0.001 | 1.533(1.276,1.843) | <0.001 |
| Categories | | | | |
| Robust | Ref |  | Ref |  |
| Pre-frail | 1.660(1.047,2.633) | 0.031 | 1.773(1.130,2.783) | 0.013 |
| Frail | 2.450(1.579,3.802) | <0.001 | 2.534(1.664,3.906) | <0.001 |
| *P* for trend | <0.001 | | <0.001 | |
| **Low CDAI** | | | | |
| Frailty status | | | | |
| Continuous | 1.421(1.140,1.772) | 0.002 | 1.516(1.211,1.897) | <0.001 |
| Categories | | | | |
| Robust | Ref |  | Ref |  |
| Pre-frail | 2.310(1.275,4.185) | 0.006 | 1.482(0.856,2.564) | 0.160 |
| Frail | 2.632(1.490,4.650) | <0.001 | 2.268(1.332,3.862) | 0.003 |
| *P* for trend | 0.004 | | 0.001 | |

For FI, 0 ≤ robust < 0.10, 0.10 ≤ pre-frail < 0.25, 0.25≤ frail < 1.

For CDAI, Low CDAI < -2.28, -2.28 ≤ Middle CDAI ≤ 1.72, High CDAI > 1.72.

Model was adjusted for age, gender, BMI, WC, SBP.

**Table S8**. Association between changes in FI and the risk of CKD after further removing extreme values of CDAI.

| **Variables** | **Model 3** | |
| --- | --- | --- |
|  | **HR [95%CI]** | ***p*-value** |
| **High CDAI** | | |
| Frailty status | | |
| Continuous | 6.183(2.958,12.927) | <0001 |
| Categories | | |
| Robust | Ref |  |
| Pre-frail | 1.913(1.133,3.230) | 0.015 |
| Frail | 1.951(1.173,3.245) | 0.010 |
| *P* for trend | 0.029 | |
| **Middle CDAI** | | |
| Frailty status | | |
| Continuous | 8.010(5.304,12.098) | <0.001 |
| Categories | | |
| Robust | Ref |  |
| Pre-frail | 1.579(1.216,2.320) | 0.002 |
| Frail | 2.370(1.740,3.228) | <0.001 |
| *P* for trend | <0.001 | |
| **Low CDAI** | | |
| Frailty status | | |
| Continuous | 7.683(4.627,12.757) | 0.002 |
| Categories | | |
| Robust | Ref |  |
| Pre-frail | 1.967(1.316,2.942) | <0.001 |
| Frail | 2.294(1.558,3.377) | <0.001 |
| *P* for trend | <0.001 | |

For FI, 0 ≤ robust < 0.10, 0.10 ≤ pre-frail < 0.25, 0.25≤ frail < 1.

For CDAI, Low CDAI < -2.28, -2.28 ≤ Middle CDAI ≤ 1.72, High CDAI > 1.72.

Model was adjusted for age, gender, BMI, WC, SBP.

**Table S9**. Subgroup analysis for the association of FI with CKD in different levels of CDAI

| **Variables** | **High CDAI** | | **Middle CDAI** | | **Low CDAI** | |
| --- | --- | --- | --- | --- | --- | --- |
|  | **HR [95%CI]** | ***p*-value** | **HR [95%CI]** | ***p*-value** | **HR [95%CI]** | ***p*-value** |
| **Age** | | | | | | |
| More than 60 | 11.6763(5.485,24.855) | <0.001 | 9.575(6.163,14.874) | <0.001 | 6.946(4.052,11.907) | <0.001 |
| Less than 60 | 34.376(5.358,220.544) | <0.001 | 56.154(18.135,173.878) | <0.001 | 84.635(24.435,293.144) | <0.001 |
| *P for trend* | <0.001 | | <0.001 | | <0.001 | |
| **Sex** | | | | | | |
| Male | 11.844(5.059,27.727) | <0.001 | 10.811(6.113,19.117) | <0.001 | 9.712(4.598,20.515) | <0.001 |
| Female | 21.572(6.082,76.508) | <0.001 | 13.274(7.251,24.300) | <0.001 | 11.286(5.771,22.071) | <0.001 |
| *P for trend* | <0.001 | | <0.001 | | <0.001 | |
| **BMI** | | | | | | |
| Normal | 12.334(5.879,25.876) | <0.001 | 10.780(6.937,16.754) | <0.001 | 10.106(5.910,17.281) | <0.001 |
| Abnormal | 35.861(3.461,371.571) | <0.001 | 27.702(8.445,86.781) | <0.001 | 15.913(4.172,60.690) | <0.001 |
| *P for trend* | <0.001 | | <0.001 | | <0.001 | |
| **MARRIAGE** | | | | | | |
| Married | 12.334(5.879,25.876) | <0.001 | 10.780(6.937,16.754) | <0.001 | 17.669(7.869,39.677) | <0.001 |
| Never married | 12.992(4.880,34.592) | <0.001 | 10.373(5.702,18.874) | <0.001 | 15.913(4.172,60.690) | <0.001 |
| Other | 16.387(0.669,401.653) | 0.087 | 9.557(1.753,52.109) | 0.009 | 17.710(4.258,73.656) | <0.001 |
| *P for trend* | 0.003 | | <0.001 | | <0.001 | |

For CDAI, Low CDAI < -2.28, -2.28 ≤ Middle CDAI ≤ 1.72, High CDAI > 1.72.

For BMI, 18.5 ≤ BMI < 24 is classified as normal, with other value deemed abnormal.

Model was adjusted for age, gender, PIR, education, marriage, drinking status, BMI, SBP and WC.

**Fig S1**. Baseline characteristics of participants with CDAI score


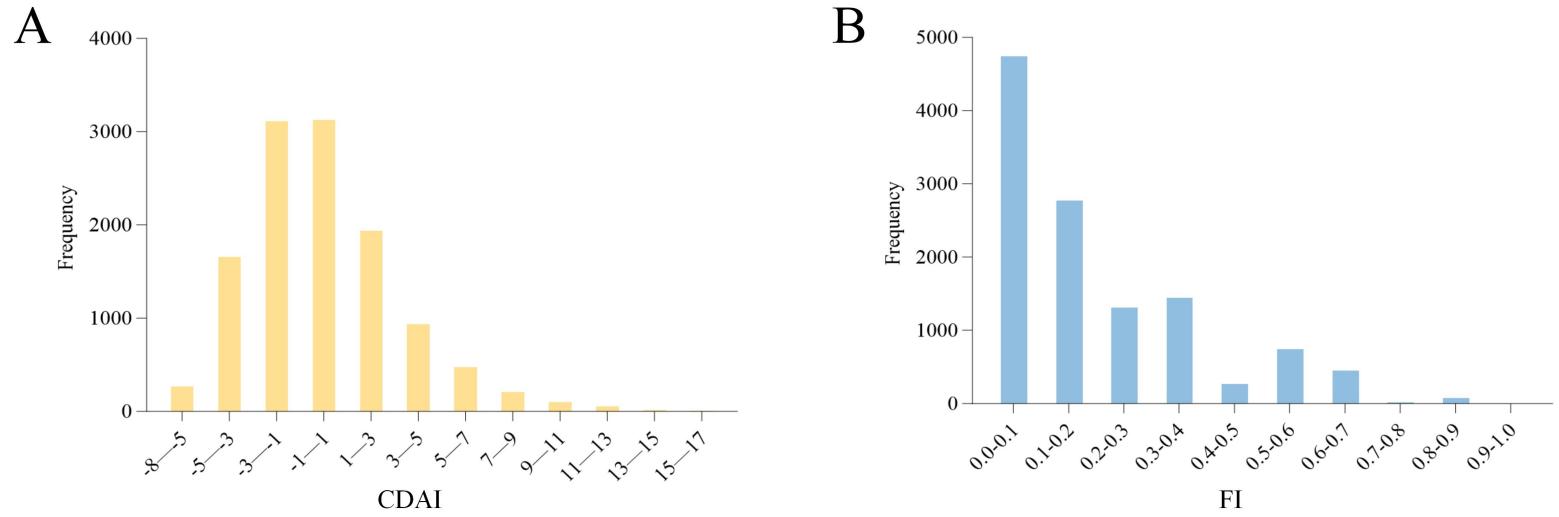


(A) Frequency distribution of CDAI score. (B) Frequency distribution of frailty index.
